# Associations Between Cognitive Profiles and Balance in Essential Tremor

**DOI:** 10.5334/tohm.969

**Published:** 2025-03-21

**Authors:** Nathan Hantke, Barbara H. Brumbach, Lauren Siegel, Martina Mancini, Delaram Safarpour

**Affiliations:** 1 Department of Neurology, Oregon Health and Science University, Portland, OR, USA; 2 Mental Health and Clinical Neuroscience Division, VA Portland Health Care System, Portland, OR, USA; 3 OHSU-PSU School of Public Health, Biostatistics and Design Program, Oregon Health and Science University Portland, OR, USA; 4 School of Graduate Psychology, Pacific University, Hillsboro, OR, USA

**Keywords:** essential tremor, cognition, neuropsychology, gait, balance

## Abstract

**Background:**

Essential Tremor (ET) is increasingly recognized as phenotypically heterogeneous disorder, which may encompass alterations in gait, balance and cognitive dysfunction. Disruption in cerebellar-thalamic-cortical circuits results in varying patterns of executive and memory dysfunction and balance disorders. The current study proposed two aims: 1) identify cognitive subtypes within individuals with essential tremor, and 2) examine for a correlation between these subtypes and gait and balance dysfunction. We hypothesize that gait and balance dysfunction are more common in individuals with ET who demonstrate greater cognitive difficulties.

**Methods:**

Seventy-one individuals underwent neuropsychological and physical therapy examinations as part of presurgical deep brain stimulation (DBS) evaluations that included measures of gait and balance (Mini-BESTest; Timed Up and Go, SARA). People with ET were categorized into Cognitively Normal (N = 29), Low Executive Function/Processing Speed (N = 17), and Low Memory Multi-domain groups (N = 25).

**Results:**

Regression analyses show that scores on the Mini-Balance Evaluation Systems Test and Scale for the Assessment and Rating of Ataxia were worse in the Low Memory and Low Executive Function groups compared to the cognitively normal group; age was also a significant predictor. Scores on the Timed Up and Go were worse for the Low Executive Function compared to the cognitive normal group; age and education were also significant predictors. Medication use was not associated with any of the clinical gait and balance tests. However, medication use and age were significant predictors of reported falls in daily life.

**Conclusions:**

A subset of individuals with ET experience cognitive dysfunction that coalesce into processing speed deficits or immediate memory deficits. These cognitive subtypes were associated with greater difficulty in balance and gait as compared to cognitively normal ET patient and this difference could not be accounted for by medications.

## Introduction

Essential tremor (ET) is one of the most common adult movement disorders, affecting 1–5% of adults over age 60 [1]. ET is recognized as a syndrome that may include isolated tremor or additional neurological features, such as cognitive impairment, language difficulties, and balance issues, in addition to tremor [2,3]. ET represents a cognitively heterogenous group, ranging from cognitively normal to dementia. While the majority of individuals with ET exhibit normal cognitive function, a mounting body of research indicates that these individuals may be more likely to develop Mild Cognitive Impairment (MCI) and dementia compared to age-matched controls [4].

Cognitive dysfunction in people with ET is thought to be associated with disruption in cerebellar-thalamic-cortical circuits, with executive dysfunction being the most observed cognitive symptom given the widespread connectivity between the thalamus and frontal cortex [5]. Specifically, individuals with ET most often show compromised performance on executive functioning tasks measuring cognitive inhibition, set-shifting, verbal fluency, and working memory [6]. Deficits in memory are also seen in individuals with ET, proposed to be related to disruption in connectivity between the hippocampus and the cerebellar-thalamic tracts, as well as the role of frontal lobe and efficient memory encoding [7,8]. Recent cross-sectional studies have attempted to characterize patterns of cognitive dysfunction in people with ET using cluster-based approaches to create cognitive phenotypes [6,7]. Results of these studies suggest that cognitive difficulties cluster mainly as memory impairment or executive dysfunction in people with ET. However, to our knowledge, these clusters have not been reproduced within different samples, and the potential relationship of these cognitive subtypes with balance and gait dysfunction have not been studied.

Impairment in balance is seven times more likely to occur in people with ET than healthy individuals [9]. This is due to the critical role of the cerebellum and associated cortical pathways in the generation and expression of both cognitive and motor abilities. Recent studies have focused towards examining a relationship between these symptoms in the ET population [10]. Executive functioning and processing speed exponentially decline throughout the aging process, particularly so in individuals with subcortical dysfunction [11]. Balance and gait and cognition are thought to share similar neural substrates [12,13]. For example, in people with Parkinson’s disease, measures of gait pace and turning have been associated with visual attention and executive functioning, while static balance has been associated with psychomotor speed and visuospatial appreciation [14]. In people with ET, number of falls, gait missteps, and balance confidence are associated with worse executive dysfunction [9,10,15]. In addition, ET has been associated with widespread cerebral atrophy in areas involved in controlling motor sequencing and voluntary motor movement, such as cerebellum, inferior and middle frontal cortices, cingulate cortices, and temporal cortex [16,17]. Cognitive impairment, particularly executive dysfunction, has been shown to be the most consistent predictor of decline in aspects of balance over time in people with ET [18].

Most studies have focused on investigating the correlation between specific cognitive tasks and different aspects of balance and gait among individuals with ET. While these findings provide insight into correlations between these two symptoms, it does not allow for examination of possible ET cognitive/motor subtypes, or examine if previously proposed cognitive subtypes are reproducible across samples. In this study, we examined the reproducibility of cognitive subtypes in individuals with ET, as well as the relationship between these subtypes and gait/balance. We hypothesized that ET subtypes with cognitive impairment (memory or executive dysfunction) would also show difficulty in gait/balance.

## Methods

The current study design was a cross-sectional, retrospective, observational study. All individuals with ET were seen between 2020 and 2023 for presurgical assessment of candidacy for Deep Brain Stimulation (DBS) at Oregon Health & Science University as part of standard clinical care. All individuals were diagnosed with ET syndrome confirmed with a clinical examination by a Movement Disorder Neurologist using the 2018 consensus statement on the classification of tremors [19]. Exclusion criteria included a diagnosis of co-occurring ET & PD (n = 2), presenting problem of cervical dystonia (n = 1), and severe psychiatric symptoms (n = 0) based upon clinical interview and self-report measures at time of evaluation.

### Neuropsychological measures

The majority of test administration was conducted via teleneuropsychology, as collection of data occurred during the initial in-person restrictions associated with the Covid-19 pandemic, with continued virtual visits offered at patient request once restrictions were rescinded for non-urgent care. The battery listed below included modified measures showing strong support of validity when utilized in teleneuropsychological services [20,21] or recommended for pre-DBS neuropsychological evaluations [22]. Normed results on cognitive measures were converted to standard scores (x̄ = 100, standard deviation = 15). A diagnosis of Mild Neurocognitive Disorder (MCI) and Major Neurocognitive Disorder (MND) were based upon DSM-5 criteria [23].

The Repeatable Battery for the Assessment of Neuropsychological Status (RBANS) Update is a cognitive screening measure which contains 12 subtests examining a variety of cognitive functions [24]. The 12 RBANS subtest raw scores were converted to age-adjusted scaled scores, which in turn created composite summary indexes based on age-adjusted normative data from the test manual. Composite indexes consist of Immediate Memory, Visuospatial/Construction, Language, and Delayed Memory. The Attention index score was not calculated due to testing limitations of the teleneuropsychology modality (Coding subtest). All patients received version Form A. Only index scores were used in the analysis, which have shown adequate internal consistency [25,26]. The RBANS has been utilized in ET, and in differentiating between cortical and subcortical presentations [27,28].

Oral Trail Making Test A (TMTA) is an oral version of the classic test of processing speed utilized for people with significant motor impairment. Individuals were asked to count from 1 to 25 as quickly as possible. Oral Trail Making Test B (TMTB) is an executive function test of mental flexibility, requiring individuals to alternate between counting and saying letters of the alphabet in numerical and alphabetical order; standard scores for oral TMT tests were based on previously published normative data for same-aged peers [29,30]. Phonemic fluency was measured via the Controlled Oral Word Associated Test (COWAT) in which individuals were asked to produce as many words as possible beginning with a specified letter during a 60-second period following parameters, with standard scores adjusted for age and education [31]. The Delis-Kaplan Color-Word Interference (CWIT) includes four conditions, providing age-adjusted scores. The first two conditions are measures of processing speed that is not dependent on motor dexterity, and the second two conditions are measures of cognitive control [32].

Beck Depression Inventory-II (BDI-II) is a 21-item self-report measure of depression symptoms. The sum of individual item scores created a composite, with higher scores reflecting greater symptom severity [33].

### Gait and Balance Performance

All individuals completed in-person gait and balance evaluations, administered by a licensed physical therapist, as part of their DBS presurgical evaluation. Testing included the following measures. 1. Mini-Balance Evaluation System Test (Mini-BESTest): a standardized balance evaluation that measures anticipatory balance, reactive postural control, sensory orientation, and dynamic gait, resulting in a total score ranging from 0 to 28 points [34]. 2.Timed Up & Go (TUG): a performance-based measure of mobility and fall risk, requiring patients to stand from a seated position, walk a set distance, turn, return to their chair, and take a seated position [35]. 3. Scale for the Assessment and Rating of Ataxia (SARA): a clinical, semi-quantitative assessment of cerebellar ataxia based on 8 categories. Total SARA scores fall between 0 (no ataxia) to 40 (most severe ataxia) [36]. 4. 360-degree turn separately for right and left, timed in seconds. Falls over the past 6 months were captured by participant self-report at the time of the presurgical evaluation.

### Defining cognitive groups

Cognitive performance in individuals with ET was categorized into five previously identified cognitive domains, consisting of memory, executive functioning, processing speed, visuospatial skills and language abilities [6]. The executive functioning domain consisted of the Oral TMTB completion time age-adjusted standard score and phonemic fluency demographic-adjusted standard score. The memory domain consisted of the RBANS Immediate Memory age-adjusted index score to allow for a direct examination of immediate memory deficits given the relatively high frequency of this impairment in ET patients [27]. Processing speed domain consisted of the oral TMTA completion time age-adjusted standard score; language domain consisted of the RBANS Language age-adjusted index score; and visuospatial domain by the RBANS Visuospatial/Construction age-adjusted index score. CWIT Inhibition was initially included in the executive functioning index, and CWIT Word Reading and Color Naming included in the processing speed index. Due to a smaller subset of patients having completed CWIT, its inclusion led to a diminished sample size. In our sample, people with ET were categorized into the same cognitive subgroup regardless of whether these additional measures were included; hence, these measures were excluded from the analysis.

Performances were defined as standard scores of 90 or greater as normal; 80–89 as low average, and 79 or lower as impaired, consistent with previously published research examining ET cognitive subtypes [6,7]. ET cognitive subtypes, as defined in previously published cluster analysis, resulted in these groups: low executive functioning, low memory multidomain, and cognitively normal [6]. Using this cluster-based subgroup criteria identified by Ratajska and colleagues [6], we first attempted to see if individuals in our sample fit the proposed profiles. For example, we determined what patients would fit into Cluster 1: memory scores in the average range, executive function and processing speed in the low range, with average visuospatial and language scores. However, at the individual level, we found less than 5 patients’ scores fit into one of the three clusters reported in Ratajska (2020). Looking closely at Ratajska and colleagues [6] results, we observed that the scores for the primary cognitive domain of each subtype (e.g., Low Memory) were driving the distinctions between clusters. We therefore used looser criteria within the primary domain to categorize our subtypes that were still consistent with the cluster analysis from Ratajska et al. (2020). Specifically, patients in our Cognitive Normal (CN) group were defined as average or better scores on cognitive domains. Patients in our executive functioning/processing speed (EF) group were defined as having low average or worse performances on either EF *or* processing speed, *and* average memory scores. The other cognitive domains did not factor in to how patients were selected into this subtype. Finally, patients with low average or worse memory scores were placed in a third group, Low Memory Multi-domain, regardless of their scores on the other cognitive domains. Interestingly, once patients were classified in this way, when looking at the group averages, the overall group profile was similar to those reported by Ratajska et al. (2020). Therefore, we suggest that these clusters are useful at the aggregate level for describing group averages, but not to classify patients at an individual level.

To assess the potential impact of delayed memory impairment on cognitive function, we constructed a delayed memory index defined by the age-adjusted RBANS Delayed Memory index score [24]. This was not included in the primary analysis given the inclusion of immediate memory and it’s hypothesized role in ET related cognitive impairment.

### Statistical analysis

Descriptive statistics were used to characterize demographic and clinical variables (Table 1), as well as the physical therapy outcome variables (Table 2). Analysis of Variance and Chi-square tests were used to assess differences in demographic and clinical variables between the cognitive subgroups. Distributions for the gait and balance outcome variables were assessed for normality. The TUG and 360 degree turn measures violated assumptions, and log transformations were performed. The transformation resolved the violations; analyses with degree turn and TUG use the log transformed value. A priori, we identified several variables to include as covariates because of their possible associations with balance outcome variables. These include age, sex, disease duration in years, years of education, and medication use.

**Table 1:** Participant demographics.


			COGNITIVE SUBTYPES		

SAMPLE (n = 71)	ALL PATIENTS	CN (n = 29)	LOW EF (n = 17)	LOW MEM (n = 25)	P*

	MEAN (SD)	MEAN (SD)	MEAN (SD)	MEAN (SD)	

*Demographics*					

Age in years	70.3 (8.7)	69.3 (9.8)	71.8 (8.7)	70.4 (7.3)	0.63

Years of education	14.8 (2.4)	15.6 (1.8)	15.3 (2.7)	13.6 (2.4)	**0.006**

Disease duration	21.6 (17.1)	23.8 (17.4)	20.6 (17.8)	19.8 (16.6)	0.66

Medication use	1.4 (1.1)	1.2 (1.0)	1.6 (1.1)	1.6 (1.1)	0.32

	n (%)	n (%)	n (%)	n (%)	

*Gender*					**0.02**

Female	33 (46.5)	10 (30.3)	13 (39.4)	10 (30.3)	

Male	38 (53.5)	19 (50)	4 (10.5)	15 (39.5)	

*Cognitive Diagnosis*					**<0.0001**

Normal	50 (70.4)	28 (56)	13 (26)	9 (36)	

MCI	19 (26.8)	1 (5.3)	4 (21.1)	14 (56)	

MND	2 (2.8)	0 (0)	0 (0)	2 (8)	


Abbreviations: MCI = mild cognitive impairment; MND = Major Neurocognitive Disorder; CN = Cognitively Normal; Low EF = Low Executive Function; Low Mem = Low Memory Multidomain. *Analysis of Variance and Chi-square were used to test for statistical significance.

**Table 2:** Average cognitive profile by cognitive subtype.


	ALL PATIENTS			COGNITIVELY NORMAL			LOW EXECUTIVE FUNCTION/PROCESSING			LOW MEMORY MULTI-DOMAIN		
			
	n	MEAN (SD)	MIN, MAX	n	MEAN (SD)	MIN, MAX	n	MEAN (SD)	MIN, MAX	n	MEAN (SD)	MIN, MAX

*Cognitive domains*												

Recent Memory	71	93.1 (17.4)	49, 126	29	^#^105.1 (9.4)	94, 126	17	^#^100.8 (10.5)	90, 123	25	*74.0 (10.4)	49, 87

Executive Function	70	98.4 (12.3)	55, 119	29	^#^103.8 (8.6)	90, 119	17	*98.8 (9.7)	80.5, 113	24	91.7 (14.7)	55, 112

Processing Speed	50	95.2 (11.2)	65, 113	20	^#^101.9 (5.5)	91, 111.5	12	*86.2 (10.6)	65, 98.5	18	93.8 (12.1)	75, 113

Language	70	97.0 (12.4)	54, 133	29	99.6 (10.0)	75, 128	17	101.1 (11.9)	80, 133	24	91.0 (13.5)	54, 122

Visuospatial	67	97.8 (15.4)	64, 131	29	101.9 (14.0)	64, 131	17	101.2 (14.7)	75, 131	21	89.5 (15.2)	66, 121

Delayed Memory	67	96.2 (15.4)	60, 122	29	101.7 (9.9)	78, 117	17	102.0 (14.7)	75, 122	21	83.9 (15.6)	60, 110


#Subtype defined by normal values in this cognitive domain.

*Subtype defined by low average or impaired in this domain (note: low EF/processing subtype was defined low scores in one or both of the domains).

Multiple regression was used to test our hypotheses that cognitive subgroups are associated with balance outcomes (Table 4). A linear regression model was assessed for each of the following balance variables: Mini-Best, Sara, TUG, 360 turning time (right and left). We also assessed self-reported number of falls using a negative binomial regression. We controlled for several covariates in each model: age, sex, disease duration in years, years of education, and medication use. Patients’ medication usage was defined as the total number of the specified medications taken by each patient on the days of the neuropsychological and balance evaluations based on electronic medical record chart review (see supplement Table 1); no individual was concurrently taking more than 4 of these medications at time of reporting. We first ran the regression models with the cognitive grouping variable and all potential covariates. Final models were run with only the cognitive grouping variable and any covariates that were found to be statistically significant in the first model; non-significant demographic covariates were removed from the final models. The exception to this is handedness which is included in the right and left degree turn final models. To correct for multiple comparisons for the five in-clinic balance variables, we applied a Bonferroni correction. Statistical significance is reported with both the original p-value and the adjusted alpha (see Table 4).

## Results

Seventy-one patients (all non-Hispanic white) met inclusion criteria and completed the neuropsychological evaluation (7 in-person visits and 64 virtual visits); Table 1.

When using adjusted criteria for ET cognitive subtypes, described above in the methods section, 29 patients met criteria of the Cognitively Normal (CN) group, 17 fit in the Low Executive Function/Processing Speed (Low EF) group, and 25 fit in the Low Memory Multidomain (Low Mem) group; Table 2. Within the overall sample, 26.8% met clinical criteria for a Mild Neurocognitive Disorder (MCI) and 2.8% for Major Neurocognitive Disorder (MND) as based upon The Diagnostic and Statistical Manual of Mental Disorders Fifth Edition [23]. There was a significant association between cognitive diagnosis and cognitive subtype (p < 0.0001), with an observed large proportion of patients with MCI or MND in the Low Mem group (Table 1).

**Figure 1:**
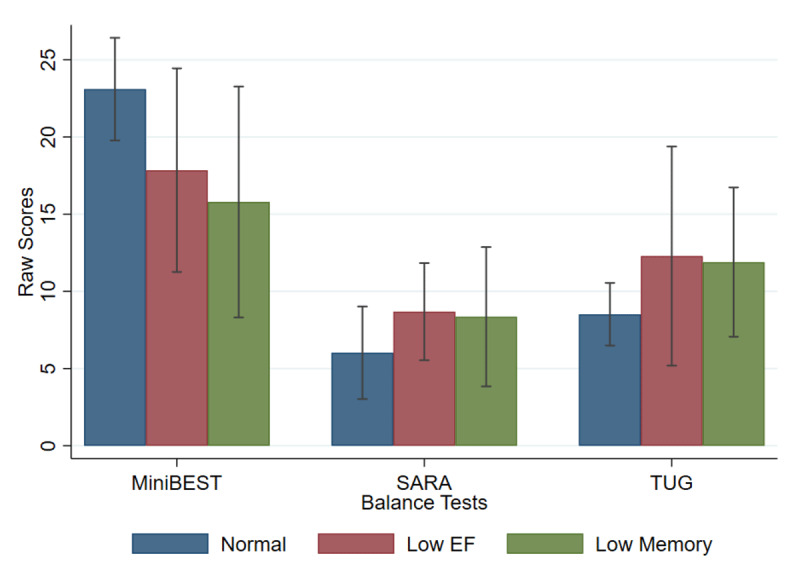
Balance performance by ET cognitive subtype, with the cognitively normal group demonstrating better performance on measures of gait and balance than the ET cognitive subgroups. Abbreviations: EF = executive function; SARA = Scale for the Assessment and Rating of Ataxia; TUG = timed up and go.

Balance and gait performance across cognitive subtype groups is presented in Figure 1 and Table 3. The following are results based on the final regression models that only include the cognitive grouping variable and covariates found to be statistically significant in the first model. After adjusting for multiple comparisons, we found a statistically significant model predicting Mini-BESTest total score (Table 4). We observed that the Mini-BESTest was worse in the Low Mem and Low EF groups compared to the CN group. Age was also a significant predictor of Mini-BESTest score, but the other covariates were not. Similarly, after adjusting for multiple comparisons, we found a statistically significant model predicting SARA total score. Both cognitive subtype and age were significant predictors. Using the log transformed TUG score, we found a statistically significant association between cognitive status subgroups and TUG time. Specifically, the TUG time was worse in the low EF group compared to CN group. Again, age was a significant predictor, in addition to education.

**Table 3:** Gait/Balance performance by cognitive subtypes groups.


	ALL PATIENTS			CN			LOW EF			LOW MEM MULT DOMAIN		
			
	n	MEAN (SD)	MIN, MAX	n	MEAN (SD)	MIN, MAX	n	MEAN (SD)	MIN, MAX	n	MEAN (SD)	MIN, MAX

*Test*												

Mini-Best	52	19.1 (6.7)	3, 28	20	23.1 (3.3)	16, 28	13	17.8 (6.6)	4, 27	19	15.8 (7.5)	3, 27

Total SARA	57	7.6 (3.8)	1, 18	20	6 (3)	2, 13.5	16	8.7 (3.1)	3.5, 12	21	8.4 (4.5)	1, 18

TUG	59	10.7 (5.1)	4.9, 35.9	22	8.5 (2)	5.2, 13.2	16	12.3 (7.1)	6.1, 35.9	21	11.9 (4.8)	4.9, 21.5

Number of falls	59	1.5 (2.4)	0, 12	22	1.1 (2.6)	0, 12	16	1.4 (1.8)	0, 6	21	1.9 (2.7)	0, 12

360 turn	55			20			16			19		

Right		3.9 (2.9)	1.4, 18		2.9 (1.5)	1.4, 8.2		4.5 (3.9)	2, 18		4.4 (2.9)	1.8, 12.3

Left		3.9 (2.9)	1.4, 19.6		3.1 (1.7)	1.4, 9.2		4.7 (4.4)	2.2, 19.6		3.9 (2.1)	1.9, 9


Abbreviations: PT = physical therapy; SARA = Scale for the Assessment and Rating of Ataxia; TUG = timed up and go.

**Table 4:** Final regression models for balance performance outcome variables.


	MINIBEST			SARA			TUG			DEGREE TURN RIGHT			DEGREE TURN LEFT				FALLS^#^		

Model																Model			

F	9.23			4.47			6.20			3.03			3.26			χ^2^	14.66		

p	0.0001*			0.007*			0.0004*			0.03			0.02			p	**0.006**		

Adj.R^2^	0.33			0.16			0.26			0.13			0.14						

n	52			57			59			55			55			n	59		

**VARIABLES**	**COEF.**	**P**	**95% CI**	**COEF.**	**P**	**95% CI**	**COEF.**	**P**	**95% CI**	**COEF.**	**P**	**95% CI**	**COEF.**	**P**	**95% CI**		**IRR**	**P**	**95% CI**

Cognitive Subtype (CN ref)																			

Low EF	–4.19	**0.04**	–8.19, –0.19	2.42	**0.04**	0.07, 4.78	0.25	**0.03**	0.03, 0.47	0.28	0.10	–0.05, 0.61	0.26	0.11	–.06, 0.58		1.26	0.62	0.50, 3.22

Low Mem.	–7.46	**<0.0001**	–10.97, –3.89	2.41	**0.03**	0.23, 4.60	0.19	0.09	–0.03, 0.42	0.32	0.04	0.01, 0.64	0.24	0.12	–.06, 0.55		2.01	0.10	0.87, 4.63

Age	–0.30	**0.003**	–0.50, –0.11	.15	**0.01**	0.03, 0.27	0.02	**0.001**	0.01, 0.03	0.02	0.01	0.005, 0.04	0.02	0.006	0.007, 0.04		1.09	**0.002**	1.03, 1.14

Education (yrs)							–0.05	**0.05**	–0.09, 0.0004										

Handedness										0.08	0.63	–0.26, 0.43	0.02	0.92	–0.32, 0.35				

Med. Number																	1.57	**0.02**	1.07, 2.31


*Indicates statistically significant after Bonferroni correction for multiple comparisons for the in-clinic PT variables (p < 0.01). #Indicates results are from a negative binomial regression. Coef. = unstandardized b coefficient. CI = Confidence Interval. IRR = Incidence Rate Ratio. CN = Cognitively Normal. Low EF = Low Executive Function. Low Mem = Low Memory Multidomain. Med Num = number of current medications.

After controlling for multiple comparisons, the models for 360 degrees turning time did not remain statistically significant.

A negative binomial regression was used to assess whether there was an association between cognitive subgroups and number of falls. While we did not find a statistically significant relationship between cognitive subgroups and number of falls (after controlling for age and medications use), we did find that medication number and age were statistically significant predictors of falls.

## Discussion

In this study, we investigated whether previously proposed cognitive subtypes in ET correlate with clinical gait and balance measures. Using a priori based cognitive domains, we found that patients only broadly classified into the ET cognitive subtypes proposed by Rajatska and colleagues [6]. A subset of patients with ET were cognitively normal, a subset demonstrated processing speed deficits, and another subset showed immediate memory deficits. In addition, we found that gait and balance were worse in those with ET and cognitive deficits.

The creation of ET cognitive subtypes (6) only worked at a group level, and individuals were not effectively binned using predetermined cognitive cut-points from prior research. While some cognitive measures used in the current study differed from those used by Ratajska and colleagues [6], the basic cognitive constructs were the same, using measures demonstrated to show good construct validity in movement disorders [27,28]. The current findings suggest that on an individual basis, people with ET likely do not fall into predictable phenotypes that can be defined using predetermined clinical variables. Rather, patients with ET as population have a tendency towards a specific constellation of cognitive difficulties that are greater than those seen in normal aging. And individuals with ET who have normal cognition demonstrate different gait and balance characteristics than those who have isolated deficits in processing speed or those that have a multidomain pattern of impairment.

Deficits in immediate memory among patients with ET have been documented in other studies examining cognitive subtypes [6,7,27]. These deficits are often thought of as difficulty with initiation of strategies for encoding information and poor immediate memory retrieval due to thalamic-frontal dysfunction with involvement of the cerebellum. This frontal-subcortical dysfunction is in contrast with declarative memory impairment involving the temporal lobes, which while also manifesting in memory deficits, is characterized by rapid forgetting [5,37]. Given the older age of the sample, it is possible that the Low Mem group reflected older adults with the early stages of co-occurring Alzheimer’s disease, or as previously hypothesized, memory deficits in ET are due to microstructural hippocampal damage [10]. However, the Low Mem group on average demonstrated worse immediate memory recall (below average) than delayed memory recall (low average), lending support to the hypothesis that memory difficulties in ET represent a unique cerebellar-thalamic-cortical process, with cognitive impairment in ET being associated with disruption in projections from the posterior cerebellum to the frontal regions [38]. These disruptions in circuits involved in refreshing and encoding information results in poor immediate memory [38]. Other studies are supportive of demonstrating dysfunction on other frontal-lobe mediated tasks in people with ET, including difficulty with staying within set and deficits with self-initiated word retrieval greater than those seen in people with PD [39]. However, the current study was not primarily designed to examine differences between immediate and delayed memory deficits in ET and this topic warrants additional research.

Inclusion in the Low EF group was primarily driven by slowed basic processing speed, as evidenced by the finding of average executive functioning within the group yet below average processing speed. This slowed speed is often attributed to medication effects in these patients. However, neither cognitive subtype nor in-clinic balance variables (excluding self-reported history of falls) were related to medication effects in this study. Slowed processing speed in particular has been documented in ET patients, with a similar hypothesized mechanism that is associated with memory and executive functioning deficits in ET, with slowed processing speed being due to cerebellar dysfunction [40,41]. The two cognitive groups composed of individuals with slowed processing speed (Low EF) and those with multidomain impairments (Low Mem) were associated with greater difficulty on most gait and balance measures as compared to patients with ET who were not experiencing cognitive difficulties. These findings are in accordance with correlations noted in PD, with gait pace showing a strong association with frontal lobe-mediated cognitive tasks and pointing to common neural circuitry shared between cognition and balance [14].

The number of reported falls were associated with advanced age and higher medication use, both of which are known risk factors for falls in older adults [42,43]. Especially given possible side effects of medications commonly prescribed for management of tremor. Even though the standardized cognitive values were age adjusted for each participant, age continued to be significant in all balance models. Falls were not associated with cognitive subtypes or other balance and gait clinical variables.

While this study provides valuable insights into the association between different cognitive profiles and balance and gait in ET, it is important to recognize its limitations. The cross-sectional design of the study captures individuals at moderate to severe tremor stage when they are considered for surgical interventions in a highly educated, white sample. While this design limits the assumptions of causality and generalizability, it captures people in a similar stage of the disease. Another limitation of this study is that most cognitive measures were administered over video, as the majority of data was collected during restrictions associated with the Covid-19 pandemic. While research suggests teleneuropsychological and in-person administrated are broadly equivalent [21], measures were administered in a non-standardized fashion which may be influenced the findings. Lastly, while most cognitive domains were based upon well-validated index measures, assessment of processing speed and executive functioning were limited due constraints of the teleneuropsychology modality and tremor in people with advanced ET, as individuals referred for DBS are at a relatively advanced state of symptom severity. However, inclusion of other well-validated measures in these domains did not adjust cognitive subtypes group membership. This suggests the current utilized measures effectively cluster patient types, and the goal of the study was to examine for an association between cognitive subgroup and balance measures, not quantitative cognitive performance.

It is important to note that the current study is utilizing data taken as part of standard clinical care in sample of patients referred for consideration of DBS and was thus not designed using an a priori power analysis. However, the findings provide guidance for future research in the characterization of ET. For example, identifying subgroups of individuals with ET who will have higher rates of gait and balance difficulties with progression of the disease is an important step for selection of the appropriate surgical intervention and appropriate counseling for possible outcomes in this population. Examination of motor-cognitive dual tasks may provide additional insight in this area and be a future direction of research.

In summary, the current findings indicate ET patients cluster into three cognitive groups with associated problems in gait/balance, and these cognitive difficulties are not due to medication effects. Specifically, individuals within the low immediate memory group exhibit challenges in gait and balance, while those with slowed processing speed demonstrated significant difficulties with gait, balance, and mobility. These findings underscore the complexity of ET as clinical syndrome, highlighting the intricate interplay between cognitive and motor dysfunction.

**Supplemental Table 1:** Medication list.


MEDICATION	FREQUENCY

Alprazolam	3

Atenolol	0

Clonazepam	2

Gabapentin	17

Lacosamide	0

Levetiracetam	0

Lorazepam	5

Metoprolol	7

Nadolol	0

Perampanel	0

Pindolol	0

Pregabalin	1

Primidone	25

Propranolol	33

Sotalol	0

Timolol	1

Topiramate	11

Zonisamide	0


## Data Accessibility Statement

The data is available on reasonable request to the corresponding author (NH), who affirms that this manuscript is an accurate and transparent account of the study reported. No important aspects of the study have been omitted.
